# Riders on the Storm

**DOI:** 10.1111/tid.14426

**Published:** 2024-12-28

**Authors:** Andrea Cona, Mario Luppi, Rosy Priya Kodiyanplakkal, Simone Perra, Alessandra Mularoni, Maricar Malinis

**Affiliations:** ^1^ Unit of Infectious Diseases IRCCS‐ISMETT Istituto Mediterraneo per i Trapianti e Terapie ad Alta Specializzazione Palermo Italy; ^2^ Section of Hematology, Department of Medical and Surgical Sciences University of Modena and Reggio Emilia, AOU Modena Modena Italy; ^3^ Division of Infectious Diseases Weill Cornell Medicine New York New York USA; ^4^ Division of Infectious Diseases Vanderbilt University Medical Center Nashville Tennessee USA

**Keywords:** HHV‐8, KICS, transplant

## Abstract

This case involves a 52‐year‐old male, who underwent a deceased donor orthotopic liver transplant 7 months prior, presented with a 2‐week history of persistent fever, anemia, thrombocytopenia, and mild elevation of liver enzymes. Upon hospital admission, the patient was febbrile, alert and oriented, hemodynamically stable. Laboratory exams revealed worsening leukopenia, anemia, thrombocytopenia, hyponatremia, and elevated ferritin. On hospital day 5, the general condition of the patient rapidly deteriorated with dyspnea, asthenia, and worsening fever and pancytopenia.Computed tomography revealed splenomegaly and minimal bilateral pleural effusion.

## 1 Case Presentation (Andrea Cona)

A 52‐year‐old male who underwent a deceased donor orthotopic liver transplant 7 months prior presented with a 2‐week history of persistent fever that failed to defervesce with antipyretics and two separate courses of antibiotics (levofloxacin and amoxicillin‐clavulanate). Outpatient blood tests showed anemia (hemoglobin 11.2 g/dL), thrombocytopenia (PLTs 48 000/mm^3^), and mild elevation of liver enzymes (AST/ALT 51/109 UI/l).

*Past medical history is as follows*. Liver transplantation from a deceased donor (October 2022) for hepatitis C (HCV)/hepatitis B and D (HBV and HDV) related liver cirrhosis complicated with hepatocellular carcinoma. He underwent HCV treatment with direct‐acting agents with sustained viral response. The patient has been maintained on long‐term entecavir for HBV. He received induction immunosuppression, basiliximab, followed by a maintenance regimen of everolimus (target trough level of 7–9 ng/mL). The pre‐transplant serologic screening revealed a mismatch with the donor for *Toxoplasma* spp. (D+/R−), while the patient resulted positive for *Cytomegalovirus* (CMV), Epstein–Barr virus (EBV), Varicella–Zoster virus (VZV), human herpesvirus 6 (HHV‐6), and herpes simplex virus 1 (HSV‐1). Serologies of donor and recipient revealed non‐detectable immunity for herpes simplex virus 2 (HSV‐2) and human herpesvirus 8 (HHV‐8). As per the protocol in our institution, a pre‐emptive approach was used for CMV disease prevention.

*The patient* lives in an apartment in Sicily (Italy) and is unemployed. He denied a recent history of rural recreational activity, intravenous drug use, and unprotected sexual intercourse. He reported contact with his brother's dog, recently diagnosed with leishmaniasis.

*Upon hospital* admission, the patient's vital signs were 38.5°C, blood pressure 120/70 mmHg, heart rate 120 beats per minute, respiratory rate 18 breaths/min, and oxygen saturation of 97% on ambient air. He was alert and oriented, hemodynamically stable; lung and heart sounds were normal on auscultation, and the rest of the physical examination was unremarkable except for palpable splenomegaly. Laboratory exams revealed worsening leukopenia (WBC 2.48 × 10^3^ uL), anemia (hemoglobin 8.1 g/dL), thrombocytopenia (PLTs 30 000/mm^3^), hyponatremia (Na 130 mEq/L), and elevated ferritin (706 ng/mm^3^), C‐reactive protein (CRP) was 97 mg/L. Liver enzymes were within normal limits. Laboratory test results are shown in Table 1.

**TABLE 1:** Initial laboratory test results.

Test	Before hospital admission	Day 1	Reference range
**WBC count (x10^3^uL)**	4.77	2.48	4.5–11
Neutrophils	3.31	1.46	1.5–7
Lymphocytes	0.81	0.51	1–3.7
Monocytes	0.74	0.42	0.2–0.9
Eosinophils	0.04	0.07	0–0.4
Basophils	0.05	0.02	0–0.1
**Platelet count (×10^3^uL)**	48	30	150–400
**Hb (g/dL)**	11.2	8.1	13.5–17.5
**Hct (%)**	33	24	41–53
**Sodium (mmol/L)**	135	130	135–145
**Potassium (mmol/L)**	4.2	3.6	3.5–5.2
**Serum creatinine (mg/dL)**	0.78	0.62	0.6–1.3
**Aspartate aminotransferase (U/L)**	51	40	15–37
**Alanine aminotransferase (U/L)**	109	67	12–78
**Ferritin (ng/mL)**	—	706	20–280
**C‐ reactive protein (mg/L)**	111	97	0–5
**lactate dehydrogenase (u/L)**	—	265	100–190

Abbreviations: Hb, hemoglobin; Hct, hematocrit; WBC, white blood cell.

On hospital day 5, the general condition of the patient rapidly deteriorated with dyspnea, asthenia, and worsening fever. Pancytopenia worsened further, and the patient required blood and platelet transfusions on a daily schedule. Computed tomography (CT) revealed splenomegaly (spleen size 17.5 cm) and minimal bilateral pleural effusion.

## 2 Differential Diagnosis for Fever and Pancytopenia After Solid Organ Transplantation (Rosy Priya L Kodyanplakkal)

The differential diagnosis of fever and pancytopenia after organ transplantation is broad and can be grouped into non‐infectious and infectious etiologies outlined in Table 2 below. Non‐infectious etiologies include adverse reactions from new medications started post‐transplant, most often related to bone marrow suppression. Some common post‐transplant medications that can cause this include valganciclovir and trimethoprim‐sulfamethoxazole. Malignancies that directly invade the bone marrow should be on the differential as well, particularly lymphomas and post‐transplant lymphoproliferative disorder (PTLD), which is most often associated with an EBV‐positive B‐cell proliferation occurring in the setting of immunosuppression. Hemophagocytic lymphohistiocytosis (HLH), characterized by an exuberant and pathologic systemic immune response, can also present this way.  HLH can be a genetic primary immunodeficiency but can also be triggered by infections such as EBV or tuberculosis or by malignancy.

**TABLE 2:** Differential diagnosis for fever and pancytopenia after solid organ transplantation.

Non‐infectious etiologies	Infectious etiologies
Malignancy Post‐transplant Lymphoproliferative disorder Lymphoma	Viral infections Parvovirus Cytomegalovirus Epstein‐Barr virus Hepatitis Human immunodeficiency virus Herpes viruses (HSV, HHV‐6, HHV‐8)
Medication induced	Tuberculosis
Hemophagocytic lymphhistiocytosis (HLH)	Fungal infections Histoplasmosis Blastomycosis Coccidiomycosis Other endemic fungi (based on donor and recipient exposures)
	Leishmaniasis
	Tick‐borne illness—consider illnesses endemic to the region where the donor and recipient are from. Examples include: Babesia Ehrlichiosis Anaplasmosis
	Donor derived infection
	Sepsis physiology

There is a broad differential for infections that can cause fever and pancytopenia. At this point, post‐transplant, a donor‐derived infection should also be considered in addition to the patient acquiring a new infection de novo. One should consider the local geography of the transplant recipient and the relevant exposures the recipient and donor may have had before the transplant.

Many viral infections can have direct cytotoxic effects on the bone marrow and cause pancytopenia. Parvovirus, EBV, and the herpes viruses (including CMV) are common culprits in the post‐transplant period. Tuberculosis and endemic mycoses like histoplasmosis can cause fever and invade the bone marrow, causing pancytopenia. *Leishmania* can cause fever and pancytopenia, but this patient did not have an apparent exposure that would make this a likely culprit for him despite the dog's history with leishmaniasis. Tick‐borne illnesses such as babesiosis, ehrlichiosis, and anaplasmosis can also cause this presentation. Still, one should also expand the differential to include tick‐borne diseases endemic to the region. Lastly, if the patient has sepsis and shock from any organism, pancytopenia can occur in addition to fevers.

## 3 Differential Diagnosis Considered by the Case Presenter (Andrea Cona)

We considered the following differentials

‐ Viral: CMV, EBV, HSV reactivation or coxsackievirus, parvovirus B19, HIV primary infection.
‐ Protozoan: Leishmaniasis, donor‐derived toxoplasmosis.
‐ Bacterial: *Brucella* spp., *Bartonella* spp., *Coxiella* spp., *Mycobacterium tuberculosis*, nontuberculous mycobacteria.
‐ Non‐infectious: EBV‐associated PTLD, HLH.

### 3.1 Clinical Course (Andrea Cona)

Blood cultures, with extended incubation for fastidious bacteria, were negative. Serological tests for HIV, *Brucella*, *Bartonella*, and *Coxiella* were performed and all resulted negative. Plasmatic viral loads for CMV, EBV, HSV‐1/2, VZV, and parvovirus B19 were negative, excluding primary infections or reactivations. Also, negative PCR on blood ruled out disseminated toxoplasmosis and leishmaniasis.

Eventually, HHV‐8 DNA on plasma was performed, resulting in positive result with over 1 million copies/mL. These findings suggest that primary HHV‐8 infection was non‐donor derived, given that HHV‐8 serology before transplant was negative for both donor and recipient.

## 4 Human Herpesvirus‐8 (HHV‐8) Epidemiology and Related Clinical Manifestations in Solid Organ Transplants (Mario Luppi)

Human herpesvirus 8 (HHV‐8) is mainly transmitted through saliva but also by sexual intercourse, by intravenous drug use or blood transfusions, and through solid organ transplants (SOT). HHV‐8 seroprevalence rates are low (3%–7%) in the United States and Northern Europe, where the incidence of HHV‐8‐related classic form of Kaposi sarcoma (KS) is low. On the contrary, HHV‐8 seroprevalence rates are higher than 50% in sub‐Saharan Africa, with the highest incidence of the endemic form of KS and about 20%–35% in the Mediterranean area and Eastern Europe, with the highest incidence of classic KS [1]. Consistent with this, HHV‐8 seroprevalence rates are higher in Southern Italy (namely 35% in Palermo, Sicily, Italy) than in Northern Italy (namely 3%–4% in Milan, Lombardia, Italy), which mirrors the different incidence rates of classic KS [2]. Of note, HHV‐8 seroprevalence rates in the United States human immunodeficiency virus‐1 (HIV‐1) positive recipients of kidney and liver transplants from HIV‐positive donors have been reported to be 38%, higher than the seroprevalence rates in the general population, raising the issue of post‐transplant KS and HHV‐8 related complications management also in the HIV SOT setting in the United States, especially in men and in men who have sex with men [3]. The risk of post‐transplant KS (PT‐KS) is 200‐500‐fold higher than in the general population and strictly dependent on the ethnic origin and/or the geographic origin of donors/recipients [4]. In a multicenter prospective study of solid organ donors and recipients from Northern‐Central Italy, the lytic immunofluorescence assay showed the best diagnostic accuracy and identified HHV‐8 seroprevalence rates of 4% and 18% in the donors and the recipients, respectively, and a seroconversion rate of 25% [5]. HHV‐8 primary infections and/or reactivations may be associated with neoplastic and non‐neoplastic disease manifestations. Among liver recipients, HHV‐8 donor‐derived transmission is more frequent, occurs earlier, and is more often associated with an aggressive clinical course. PT‐KS is the most common HHV‐8‐related disease after SOT, more frequently occurring within a median of 13 months after transplantation. It involves the skin in most cases but is also visceral in 10% of the cases [6]. In liver transplant patients, PT‐KS is visceral in about 50% and mortality up to 60% of the cases [6]. HHV‐8‐related lymphoproliferative diseases, including primary effusion lymphoma (PEL) (including the solid extra cavitary variant of PEL), diffuse large B‐cell lymphoma, and multicentric Castleman disease (MCD) of plasma cell type are sporadic in the SOT setting, while resulting more frequent in the HIV infected population [1, 4]. Of note, in HIV‐positive individuals, the definition of an MCD flare/attack includes fever, elevated levels of CRP (higher than 2 gr/dL), which is a surrogate marker of interleukin‐6 (IL‐6), and at least three out of a variety of clinical signs and symptoms, including lymphadenopathies, splenomegaly, pleural effusions, ascites, jaundice, edema, rash, cerebral neurologic symptoms, cough, nasal obstruction, xerostomia, and autoimmune hemolytic anemia [1]. That's why the exclusion of MCD upon histologic examination of lymphadenopathy, if present, is mandatory to confirm a diagnosis of KS‐associated inflammatory syndrome (KICS) due to the similarities between the two diseases [7]. The working case definition of KICS was provided by Polizzotto et al. in HIV‐positive individuals and required at least two clinical manifestations from at least two of the three categories represented by clinical symptoms, like fever, fatigue, edema, cachexia, respiratory symptoms, gastrointestinal disturbances, arthralgia and myalgia, altered mental state, and neuropathy with or without pain; laboratory abnormalities like anemia, thrombocytopenia, hypoalbuminemia, hyponatremia and imaging abnormalities, like lymphadenopathy, splenomegaly, hepatomegaly, and body cavity effusions. In addition, CRP levels higher than 3gr/dL and extremely high HHV‐8 viral load (more than 1000 copies/mL in plasma) are required for a final diagnosis of KICS [7]. Of note, all the first 10 KICS patients described by Polizzotto et al. had KS, and two also had PELs [7]. Mularoni et al. described the first case of KICS in a liver–kidney transplant patient, showing a constellation of clinical symptoms with laboratory and imaging abnormalities consistent with the working case definition of KICS [8]. Treatment with foscarnet and 4 weekly doses of rituximab led to the disappearance of fever, negative HHV‐8 DNA, and normalization of both blood cell counts and CRP/IL‐6 levels [8]. A case of simultaneous occurrence of KS of lymph nodes and KICS in an Italian heart transplant recipient with primary donor‐transmitted HHV‐8 infection was also reported [9]. As we have now recognized KICS as a new clinicopathologic entity both in HIV and SOT settings, we have reviewed the past literature and found case descriptions of HHV‐8‐associated nonmalignant illnesses, which may now be classified as KICS. We first provided the formal proof of HHV‐8 donor transmission from an HHV‐8 seropositive donor to two HHV‐8 seronegative twin kidney recipients [10]. Four months after renal transplantation, one recipient developed disseminated KS and HLH, who recovered following pegylated liposomal doxorubicin and foscarnet. In contrast, the other recipient developed a clinical syndrome characterized by high fever, splenomegaly, bone marrow failure, and peripheral pancytopenia. The latter was treated with antivirals and intravenous immunoglobulins but died of multiorgan failure (MOF) in 1 month [10]. The possibility is raised that this was the first case of KICS, ante litteram. In the French nationwide multicenter prospective study, including kidney recipients undergoing transplantation between October 2000 and December 2003 and reporting an incidence of PT‐KS of 13% following HHV‐8 reactivation, 64 cases of HHV‐8 donor‐derived infections were also reported [11]. Three of these cases developed an HHV‐8‐related disease, namely PT‐KS in two, and a nonmalignant clinical syndrome characterized by fever, hepato‐splenomegaly, pancytopenia, and high‐level HHV‐8 viremia, which resolved following rituximab therapy [11, 12]. This case of HHV‐8‐associated non‐neoplastic disease could now be classified as KICS. Similarly, out of five cases of HHV‐8 primary infections in a series of liver transplant patients from Northern Italy, two developed nonmalignant illnesses resembling KICS [13]. Both patients were treated with cidofovir only and died in 2–3 months of MOF. In a multicenter study of transplant patients from Northern‐Central Italy, one death for MOF was reported, despite antiviral treatment, due to a nonmalignant disease characterized by fever, pancytopenia, and effusions, which was likely to be another case of KICS [5]. Thus, the occurrence of KICS may be yet largely unreported in the SOT setting, and future studies in geographic areas endemic for HHV‐8 infection will hopefully give insights into the true incidence of HHV‐8‐related nonmalignant illnesses and their most appropriate management. It is conceivable that, as in the case of PT‐KS, whenever possible, immunosuppression regimens based on calcineurin inhibitors (CNI) should be switched to mTOR inhibitors, like everolimus and sirolimus [4]. In addition to its antiproliferative effects, sirolimus allows the early recovery, within 7–10 days from the switch from a CNI, of HHV‐8 specific T‐cells, both naive and central memory T‐cells, producing interleukin‐2 (IL‐2) only and both IL‐2 and interferon‐gamma, contributing to PT‐KS resolution [4].

### 4.1 Clinical Course (Andrea Cona)

At our center, ISMETT (Palermo, Italy), there is an active protocol to pre‐emptively monitor HHV‐8 infections in recipients with HHV‐8 positive serology (R+) and recipients with mismatch (D+/R−) and treat HHV‐8‐related diseases. Since 2011, HHV‐8 serology (IFA lytic and latent) on all donors and recipients. In patients with an intermediate or elevated risk of HHV‐8 reactivation/infection (R+ and D+/R−), HHV‐8 DNA on blood is performed monthly, and strict clinical follow‐up is provided. Since 2017, a protocol for prompt treatment of patients with detectable viremia was implemented with an early switch to mTOR inhibitors (with or without antiviral). Patients with KICS receive Anti‐CD20 monoclonal antibody (rituximab).

Unfortunately, the patient in this case discussion was not included in the monitoring protocol since the pre‐transplant serologies of both recipient and donor were negative (D−/R−). However, our experience with this infection leads us to have high clinical suspicion in recipients with unexplained fever and pancytopenia and to include HHV‐8 infection among differential diagnoses promptly.

According to Polizzotto criteria [17], KICS was diagnosed due to the presence of the following signs/symptoms: fever, fatigue, respiratory symptoms, anemia, thrombocytopenia, hyponatremia, splenomegaly, and body cavity effusions plus the presence of elevation of CRP and HHV‐8 elevated viremia. KICS is a non‐neoplastic HHV8‐related clinical manifestation characterized by fever, hyperinflammatory host responses, bone marrow failure, and organ damage. Moreover, inflammatory markers and liver enzymes were persistently elevated (CRP 139 mg/L, ferritin 1157 ng/mL, LDH 313 u/L, AST/ALT 120/107 u/L), suggestive of a “cytokine storm.”

Regarding therapeutic options, several antivirals, including ganciclovir, foscarnet, and cidofovir, inhibit HHV‐8 replication in vitro [15, 16]. However, there is limited experience in vivo^4^. Foscarnet may have a role in HHV‐8‐related inflammatory diseases and HHV‐8‐associated hemophagocytosis [17, 18, 19]. Rituximab represents an emerging and promising adjunctive treatment for KICS [7, 8, 12, 20].

We initiated human immunoglobulins and foscarnet that needed to be suspended after 7 days due to adverse effects. HHV‐8 viremia at this point was 1 700 000 copies/mL. To modulate the HHV‐8‐associated cytokine storm, after discussion with a hematologist expert in the field, we decided to start a 4‐dose cycle of rituximab with some clinical benefits.

On hospital day 30, the poor clinical condition of the patient, along with the persistent pancytopenia and elevation of inflammatory markers (Table 3), despite ongoing therapy, raised concerns of unregulated hyperinflammation suggestive of HLH.

**TABLE 3:** Laboratory test results during hospital stay.

Test lab	Day 5	Day 30	Reference range
**WBC count** (×10^3^uL)	2.66	2.17	4.5–11
**Platelet count** (×10^3^uL)	25	9	150–400
**Hb** (g/dL)	6.6	5.9	13.5–17.5
**Ferritin** (ng/mL)	706	1426	20–280
**C‐ reactive protein** (mg/L)	97	155	0–5
**lactate dehydrogenase** (u/L)	265	570	100–190

Abbreviations: Hb, hemoglobin; WBC, white blood cell.

## 5 Clinical Management of the Recurrence of HHV‐8‐Related Hyper‐Inflammatory State (Mario Luppi)

Despite the switch from CNI to everolimus, a course of foscarnet (although shorter than initially planned because of toxicity), and two doses of rituximab, the patient's clinical course worsened, HHV‐8 DNA load increased, and the patient's conditions deteriorated, suggesting the recurrence of a hyper‐inflammatory state. The possibility is raised that HHV‐8 has triggered HLH. In liver recipients, the occurrence of HLH has already been described to be triggered mainly by viruses, including not only HCV and beta‐herpesviruses, like human herpesvirus‐6 and CMV, but also gamma‐herpesviruses, namely EBV, and HHV‐8 [14]. It should be noted that the findings in the patient's peripheral blood were nonspecific, while the absence of signs of hemophagocytosis in the bone marrow does not rule out HLH. Based on the recommendations by the HLH steering committee, a diagnosis of HLH is confirmed if five of eight parameters are present, including fever, splenomegaly, cytopenia, affecting at least two lineages, hemophagocytosis signs in bone marrow, spleen and/or lymph nodes, hypertriglyceridemia, hypofibrinogenemia, low or absent NK‐cell activity, hyperferritinemia, and increased soluble IL‐2 receptor [15]. A tool to calculate the probability of HLH should be used [22, 23], and if the resulting score applied is more than 90%, the likelihood of HLH in this case would be extremely high, guiding treatment. Consistent with this, the treatment algorithm for adult patients with EBV and/or HHV‐8‐associated HLH should include rituximab and antivirals. Measures to control hyperinflammation should be employed, including high‐dose corticosteroids, like dexamethasone (10 mg/m^2^ daily for 2 weeks as initial doses, then 5 mg/m^2^ for 2 weeks, 2.5 mg/m^2^ for 2 weeks, 1.25 mg/m^2^ for 1 week, and taper and discontinue in 1 week) and intravenous immunoglobulins (up to 1.6 g/kg in split doses over 2–3 days), following the modified HLH‐94 protocol [15]. The simultaneous/subsequent occurrence of KICS and HLH following primary HHV‐8 infection has already been reported [8]. Studies assessing interleukin‐6/interleukin‐10 and other cytokine signatures are warranted to characterize better the spectrum and distinct features of KSHV/HHV‐8‐related non‐neoplastic diseases in SOT recipients and better differentiate between HLH and KICS.

### 5.1 Clinical Course (Andrea Cona)

*H* Score was applied, resulting in 257 with a probability of HLH of > 99% [15] (Table 4). Bone marrow aspirate and biopsy were performed. The aspirate material was inconclusive, and no HLH patterns were found, but HHV‐8 DNA on bone marrow aspirate was > 8 million copies/mL. No hematological alteration suggests lymphoproliferative disease or megakaryocytes were found during the biopsy examination.

**TABLE 4:** *H* Score application.

Criteria	Value	Score
**Known underlying immunosuppression**	Yes	+ 18
**Temperature, °C**	38.4–39.4	+ 33
**Organomegaly**	Hepatomegaly or splenomegaly	+ 23
**Number of cytopenias**	3 lineages	+ 34
**Ferritin, ng/mL**	2,000–6,000	+ 35
**Tryglicerides, mg/dL**	> 354	+ 64
**Fibrinogen, mg/dL**	≤ 250	+ 30
**AST, u/L**	≥ 30	+ 19
**Hemophagocytosis features on bone marrow aspirate**	No	0
**Total**	**> 99% probability of HLH**	256 points

Abbreviations: AST, aspartate aminotransferase; HLH, hemophagocytic lymphohistiocytosis.

We then decided to start a high dose corticosteroid therapy, as suggested by La Rosée et al., with 16 mg (10 mg/m^2^) for 2 weeks, followed by a tapering protocol every 2 weeks for a total of 2 months of corticosteroid therapy [15].

**FIGURE 1:**
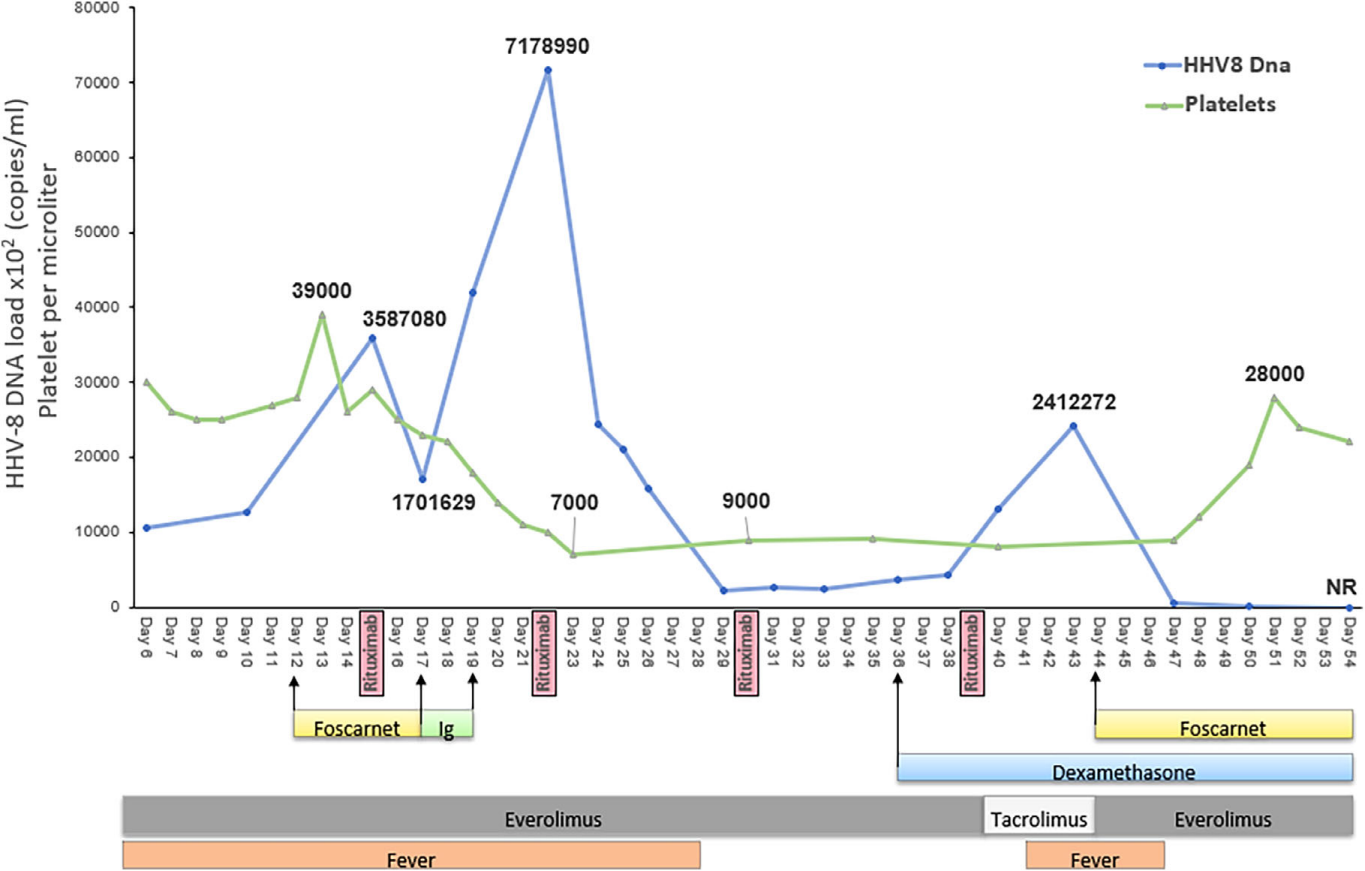
The clinical course of the patient and the therapies received.

Given the high viral load in bone marrow and peripheral blood, we decided to restart foscarnet treatment. Viremia dropped, and the patient's general conditions improved in the following 72 h with the normalization of inflammatory markers and transaminases. Therapy with foscarnet was continued for 21 days with good renal tolerance. By day 61, platelets had increased above 20 000/mm^3^, and the general condition was good. We decided to suspend foscarnet once HHV‐8 DNA viremia was undetectable. Due to clinical stability, the patient was discharged with a tight monitoring schedule (Figure 1, Video S1: https://tts.org/component/tts/?view=presentation&id=207321&Itemid=134).

## 6 Conclusion

This case highlights the importance of recognizing HHV‐8 infection, which could be catastrophic if not identified in time. SOT recipients with unexplained fever should be evocative of this in endemic and non‐endemic regions.

Untreated HHV‐8‐related diseases can cause severe non‐neoplastic disease in solid organ transplant recipients. Few data are currently available on the appropriate management of this infection, and no specific therapy is known, often relying on the introduction of mTOR inhibitors for HHV‐8 infection and antivirals/immunomodulatory agents for managing KICS.

Further studies are needed to characterize the spectrum of HHV‐8‐related non‐neoplastic diseases better and to define the optimal therapeutic approach for these syndromes.

## Author Contributions

**Andrea Cona**: conceptualization, data curation, writing–original draft. **Mario Luppi**: writing–original draft. **Rosy Priyandakal**: writing–original draft. **Simone Perra**: writing–review and editing. **Alessandra Mularoni**: writing–review and editing. **Maricar Malinis**: supervision, conceptualization, writing–review and editing.

## Conflicts of Interest

The authors declare no conflicts of interest.

## Supporting information



Video recording of clinicopathologic conference– Riders on the storm.

## Data Availability

Data available on request from the authors
